# Impact of informal care on the mental health of caregivers during the COVID-19 pandemic

**DOI:** 10.1093/pubmed/fdad193

**Published:** 2023-10-02

**Authors:** Ludmila Fleitas Alfonzo, Yamna Taouk, Eric Emerson, Tania King

**Affiliations:** Centre for Health Equity, Melbourne School of Population and Global Health, The University of Melbourne, Carlton, Melbourne, VIC 3053, Australia; Centre for Health Equity, Melbourne School of Population and Global Health, The University of Melbourne, Carlton, Melbourne, VIC 3053, Australia; Centre for Disability Research, Faculty of Health and Medicine, Lancaster University, Lancaster LA1 4YW, UK; Centre for Disability Research and Policy, Faculty of Health Sciences, University of Sydney, Sydney, NSW 2141, Australia; College of Nursing and Health Sciences, Flinders University, Adelaide, SA 5042, Australia; Centre for Health Equity, Melbourne School of Population and Global Health, The University of Melbourne, Carlton, Melbourne, VIC 3053, Australia

**Keywords:** GHQ-12, psychological morbidity, SARS-CoV-2, unpaid caregiving

## Abstract

**Background:**

Informal care can affect the mental health of caregivers. The COVID-19 pandemic precipitated many people into informal care. Little is known about the longitudinal effect of informal care throughout the pandemic. We investigate changes in mental health in relation to changes in informal care between July 2020 and September 2021.

**Methods:**

Using data from Understanding Society, we applied fixed-effects modelling to assess mental health variations associated with changes in caregiving among 13 557 participants (50 430 observations). Hours of weekly care were categorized as 0, 1–19, ≥20. Mental health was measured using the General Health Questionnaire (GHQ-12) as a continuous score and a binary indicator. Main analyses were stratified by gender.

**Results:**

Compared to when delivering 0 hours care/week, the GHQ-12 scores of women providing care for 1–19 hours/week were 0.46 points higher (95%CI: −0.11, 1.09), while their mental health scores were 0.99 higher (95%: 0.08, 1.90) when caring for ≥20 hours/week. Changes on the binary GHQ-12 measure were only evident for women when providing ≥20 hours of weekly care. These changes were not substantial among men.

**Conclusion:**

Informal care adversely impacted the mental health of women carers during the COVID-19 pandemic. Support programmes for informal carers should focus on alleviating caregiving loads in women.

## Introduction

Informal carers provide unpaid assistance to a friend or a relative in need of regular support due to long-term conditions, including physical or mental illness, a disability, problems related to alcohol/substance use and old age.^1^ Between 2018 and 2019, 7% (9.1 million people) of the UK population were undertaking informal caring roles.^2^ The COVID-19 pandemic has increased the need for care, precipitating many people into informal caring roles.^3^ It is estimated that ~4.5 million people entered new caring roles, with the number of informal carers in the UK growing from 9.1 in 2019 to 13.6 million in 2020.^4^

Pre-pandemic estimates suggest that informal care can impact the mental health of caregivers.^5–7^ A systematic review investigating the causal effect of caring for older family members found that, on average, these roles led to a high prevalence of depressive symptoms and poor general mental health.^8^ Longitudinal research in the UK and Netherlands shows a similar trend, suggesting that informal care predicts poor psychological health among people who support family members with long-term conditions.^7^ These findings were also replicated across 11 European countries.^6^

The COVID-19 pandemic and the closure of non-essential services led to high levels of distress in the general population.^9^ Emerging longitudinal research indicates that the mental health decline was more significant among informal carers than in their non-caring counterparts.^10–12^ Using the UK household survey, evidence from early stages of the pandemic showed that the mental health decline in the population was larger for informal carers than among their non-caring counterparts.^10^^,^^12^ Whitley et al. (2021) found that while the mental health of non-carers improved in July 2020, when COVID-19-related restrictions were relaxed, the mental health of informal carers remained low, this finding highlights the need of exploration of the mental health impact of informal care further into the later stages of the pandemic. Only one previous paper assessed mental health changes attributed to informal care between 2020 and 2021 in the UK.^11^ The study conducted by Costi et al. (2023) showed that while the mental health decline of informal carers who provided care to someone outside their households fluctuated across different levels of restrictions, the mental health of informal carers was consistently worse than that of non-carers over the course of the pandemic.

Many theories have been proposed to explain the mental health effects of informal care.^13^^,^^14^ One theory, the role adaptation theory, holds that mental health constrains are higher at the start of caregiving when people need to adapt to their new role.^13^ This transition point is also when informal carers are in the highest need of support.^15^ Support services, however, were limited during the lockdowns,^16^ with many services being closed or moved online.^4^ Another explanation is related to the family effect of chronic illness.^17^ Informal carers deeply care about their caring recipients, worrying that the health of their loved ones may worsen.^17^^,^^18^ Although, not directly related to the caring role, this persistent state of distress amplifies the mental health strain of caregivers.^17^^,^^18^ During the pandemic, informal carers faced considerable concerns, since many of the individuals they were caring for were at high risk of developing life-threatening symptoms of COVID-19.^4^^,^^16^ Therefore, it is feasible that the uptake of caring demands during this time may have exacerbated the existing mental health effects of informal care.

Building on existing evidence from Understanding Society (a UK longitudinal household study), this study aims to extend the current evidence base by assessing within-person changes in mental health outcomes of informal carers from July 2020 to September 2021. Since the mental health impact of informal care among out of home caregivers has been established using this sample,^11^ we focus this paper on the provision of home care—that is, those who were caring for someone living within their household. In addition, we disaggregate caregivers according to hours of weekly care, to explore the mental health effects of informal care among intense caregivers (those caring for ≥20 hours/week) and non-intense caregivers (caring for 1–19 hours/week). Lastly, acknowledging the gender differences in the mental health effects of informal care,^5^ we present a gender-stratified analysis of this association.

## Methods

### Participants

This paper uses longitudinal data from five waves of the Understanding Society COVID-19 survey: July 2020, November 2020, January 2021, March 2021 and September 2021. Understanding Society is a large and representative panel survey of UK households.^19^ The overall response rate of the COVID-19 survey was low. We analysed data from participants aged 16 years and over, for whom data on informal care were sought. See Supplementary Methods for sample details.

### Measures

#### Exposure: informal care

A three-category variable was derived to reflect caregiving intensity: (i) 0 hours of weekly care, (ii) 1–19 hours weekly and (iii) 20 hours or more. Care was defined as being provided to someone sick, disabled or elderly and living in the same house as the carer (see Supplementary Methods).

#### Outcome: mental health

Mental distress was measured using the General Health Questionnaire (GHQ-12), a validated measure of psychological morbidity widely used in population-based surveys including the Health Survey for England^20^ and as a screening tool for common mental health disorders.^21^ We derived two mental health measures: a continuous measure (0–36, higher scores indicating poorer mental health) and a binary measure of GHQ caseness (see Supplementary Methods).

#### Covariates

Time invariant confounding factors were accounted for by our analytical model. The following time-varying covariates were included in models: age (continuous measure), living with a partner (yes/no), living with children <5 years (yes/no) and living with an elderly relative (yes/no). All analyses were stratified by gender (see Supplementary Methods).

Exposure to informal care, the mental health outcome and all included covariates were measured across all included COVID-19 surveys.

### Statistical analysis

All analyses were conducted using Stata 16. We investigated the feasibility of a fixed-effects analysis by examining changes in caregiving across time and using the xttab and xttrans commands in Stata (Supplementary Tables 1 and 2). We assessed the distribution of socio-demographic characteristics, relevant covariates and the mental health outcome according to caring status. Fixed-effects longitudinal regression models were fitted to estimate changes in mental health in relation to changes in informal care separately for men and women, with individual respondents specified as the panel variable and wave as the time variable—see Supplementary Methods for details.^22^ We restricted the analytical sample to participants who did not display clinical levels of mental distress (GHQ caseness = 0) in wave 4 (July 2020) to ensure that mental health changes in GHQ-12 reflect variations in caregiving after the start of the study period. All analyses were weighted using longitudinal weights provided in Understanding Society and repeated in a non-stratified sample (gender combined).

### Missing and non-response

**Fig. 1:**
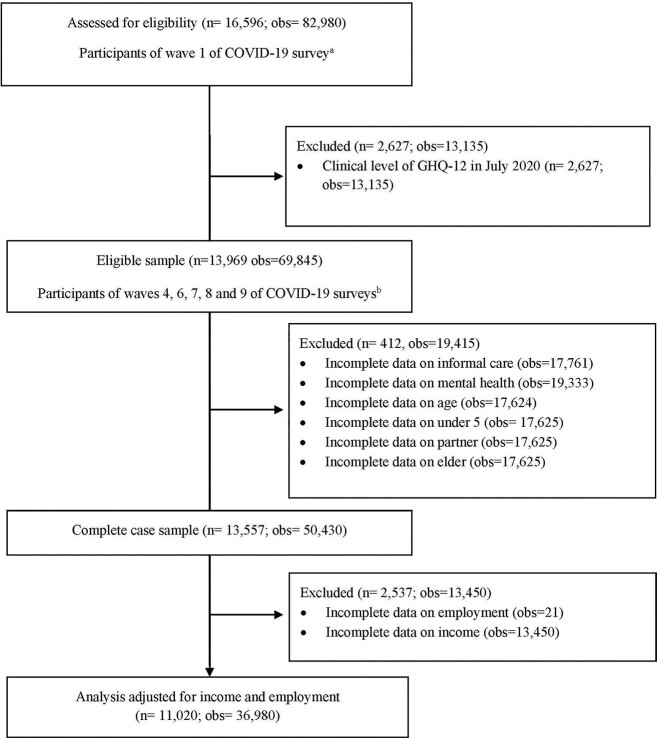
**Participants flowchart.** Notes: ^a^July 2020. ^b^November 2020, January 2021, March 2021 and September 2021.

Out of 16 596 participants who took part on wave 1 (April 2020) of the COVID-19 survey, 13 969 (69 845 observations) respondents, who participated in waves 4, 6, 7 and 9 of the COVID-19 survey, were eligible for our analyses. After excluding observations with incomplete data, the final sample included 13 557 unique respondents with 50 420 observations. Figure 1 displays the flow of participants through the eligible sample.

### Multiple imputation

Multiple imputations were carried out using chained equations with 100 imputations (see Supplementary Methods).

#### Sensitivity tests

Sensitivity analyses were performed to control for within person changes in income and employment. These were not included in main models as we judged that these factors could plausibly be on the causal pathway between informal care and mental health, and thus their inclusion in models as confounders could bias estimates. Employment was categorized as employed, unemployed/not in labour force. Quintiles of total weekly household income were derived from total weekly household income after tax deductions. All analyses were repeated on an imputed sample.

## Results

A total of 50 420 observations were included in the complete case analyses. Figure 1 displays the rates of attrition for the eligible sample. Table 1 displays the characteristics of participants in the sample. Participants contributed to an average of four waves of data (mean: 4.38 SD: 1.11), ranging from two to five waves. There were slightly more women than men participating in the COVID-19 surveys and women were overrepresented as caregivers, particularly among those providing care for 20 hours or more. Informal carers were more likely to be unemployed and live in households with lower incomes than non-carers. Informal carers were also more likely to live with an elderly person and without a partner.

**Table 1:** Population weighted socio-demographic characteristics of participants from Understanding Society COVID-19 surveys

	Hours of informal care			All
	0 hours/week obs = 47 839	1–19 hours/week obs 1202	≥20 hours/week obs = 1389	*n = 13 557, obs = 50 430*
**Outcome**	**%**	**%**	**%**	**(%)**
GHQ-12 scores (mean (SD))	11.0 (4.78)	11.8 (5.06)	12.1 (5.33)	11. 1 (4.80)
GHQ-12 caseness				
No	86.7	83.6	82.0	86.5
Yes	13.3	16.4	18.0	13.5
**Covariates**				
Age				
15–24	9.25	11.5	7.82	9.26
25–44	27.8	20.4	20.4	27.3
45–64	36.6	40.2	41.7	36.8
65+	26.4	27.8	30.2	26.6
Gender				
Women	51.1	44.6	60.0	51.1
Men	48.9	55.4	40.0	48.8
Ethnicity				
White UK	88.1	80.6	84.9	87.8
Other	11.1	19.4	15.1	12.2
Employment/occupation				
Employed	61.0	57.4	31.3	60.0
Unemployed	39.0	42.6	68.7	40.0
Household income(quintiles)				
1 (lowest)	22.5	23.5	27.6	22.7
2	19.9	18.2	30.6	20.2
3	19.2	23.8	25.8	19.5
4	18.2	17.6	9.37	17.8
5 (highest)	20.3	17.0	6.62	19.9
Living with partner				
Yes	63.9	68.0	77.9	64.5
No	36.1	32.0	22.1	35.5
Child under 5 years				
No	92.4	96.4	89.9	92.4
Yes	7.58	3.56	10.1	7.56
Living with an elderly relative				
No	86.3	60.1	68.0	85.1
Yes	13.7	39.9	32.0	14.9
Wave				
4 (July 2020)	21.4	22.9	20.1	21.4
6 (November 2020)	18.5	17.8	17.0	18.4
7 (January 2021)	18.6	18.4	18.9	18.6
8 (March 2021)	20.4	21.3	21.3	20.5
9 (September 2021)	21.2	19.5	23.3	21.2

Note: mean and weighted percentages using wave 9 population weights. Number of participants (*n*) and observations (obs) based on complete case sample distribution.

### Fixed effects regression analyses

Tables 2 and 3 show fixed-effects regression results for women and men, respectively. Changes in continuous scores of GHQ-12 related to changes in caregiving were only observed for women. When caring 1–19 hours/week women showed a 0.46 (95%CI: −0.11, 1.09) increase in GHQ-12 scores compared to when providing 0 hours of care, while caring for ≥20 hours/week was associated with 0.99 increased of mental health scores (95%CI: 0.08, 1.90). For men, informal care was associated with an increase of 0.20 (95%CI: −0.66, 1.05) of GHQ-12 scores when providing 1–19 hours of care/week, changes were similar when caring for ≥20 hours/week.

**Table 2:** Mental health effects of within person changes in informal care for women

	Model 1	Model 2
	*n = 6126, obs = 20 133*	*n = 6126, obs = 20 133*
**GHQ-12 (scores)**	**β (95%CI)**	**β (95%CI)**
0 hours of care	*ref*	*ref*
1–19 hours of care	0.46 (−0.11, 1.09)	0.67 (−0.13, 1.47)
≥20 hours of care	0.99 (0.08, 1.90)	0.89 (−0.31, 2.09)
**GHQ-12 (caseness)**	**OR (95%CI)**	**OR (95%CI)**
0 hours of care	*ref*	*ref*
1–19 hours of care	1.29 (0.73, 2.32)	1.68 (0.79, 3.56)
≥20 hours of care	2.22 (1.12, 4.43)	1.99 (0.83, 4.77)

Model 1: adjusted for age, household structure (living with a partner, children aged < 5 years and living with an elderly) and wave of COVID-19 survey.

Model 2: adjusted for variables in Model 1, employment and quintiles of weekly household income.

**Table 3:** Mental health effects of within person changes in informal care for men

	Model 1	Model 2
	*n = 5850, obs = 21 980*	*n = 5627, obs = 15 618*
**GHQ-12 (scores)**	**β (95%CI)**	**β (95%CI)**
0 hours of care	*ref*	*ref*
1–19 hours of care	0.20 (−0.66, 1.05)	0.30 (−0.75, 1.35)
≥20 hours of care	0.21 (−0.51, 0.93)	−0.33 (−1.15, 0.48)
**GHQ-12 (caseness)**	**OR (95%CI)**	**OR (95%CI)**
0 hours of care	*ref*	*ref*
1–19 hours of care	0.91 (0.42, 2.01)	0.85 (0.32, 2.29)
≥20 hours of care	0.74 (0.28, 1.92)	0.51 (0.15, 1.68)

Model 1: adjusted for age, household structure (living with a partner, children aged <5 years and living with an elderly) and wave of COVID-19 survey.

Model 2: adjusted for variables in Model 1, employment and quintiles of weekly household income.

When considering GHQ caseness, evidence of an association was only present for women when providing ≥20 hours of weekly care. In comparison to when not caregiving, odds of clinical levels of mental distress among women were 2.22 (95%CI 1.12, 4.43) times higher when caring for ≥20 hours/week.

Fixed-effects regressions for all participants (gender combined) are shown in Table 4 and described in the Supplementary Results file.

**Table 4:** Mental health effects of within person changes in informal care for all participants

	Model 1	Model 2
	*n = 13 557, obs = 50 430*	*n = 11 020, obs = 36 980*
**GHQ-12 (scores)**	**β (95%CI)**	**β (95%CI)**
0 hours of care	*ref*	*ref*
1–19 hours of care	0.36 (−0.18, 0.90)	0.54 (−0.14, 1.22)
≥20 hours of care	0.77 (0.12, 1.43)	0.55 (−0.32, 1.42)
**GHQ-12 (caseness)**	**OR (95%CI)**	**OR (95%CI)**
0 hours of care	*ref*	*ref*
1–19 hours of care	1.21 (0.75, 1.94)	1.32 (0.71, 2.47)
≥20 hours of care	1.75 (0.98, 3.14)	1.49 (0.72, 3.07)

Model 1: adjusted for age, household structure (living with a partner, children aged <5 years and living with an elderly) and wave of COVID-19 survey.

Model 2: adjusted for variables in Model 1, employment and quintiles of weekly household income

### Sensitivity analysis

Although results from sensitivity analyses did no vary substantially, our estimates provided high uncertainty levels when accounting for changes in employment and quintiles of weekly household income. With not caring as reference, women had a 0.67 (95%CI: −0.13, 1.47) increase in mental health scores when caring for 1–19 hours; these scores were 0.89 points higher when caring for ≥20 hours/week. When assessing GHQ caseness, women displayed 1.99 higher odds of poor mental health (95%CI: 0.83, 4.77) when caregiving for ≥20 hours. All our results were consistent across the imputed analyses (see Supplementary Tables 3–5).

## Discussion

### Main findings of this study

We found that the mental health of informal carers during the COVID-19 pandemic was worse when caregiving. Mental health changes were only evident for women, more significantly for intense caregiving. We observed substantial gender differences in GHQ-12 caseness, with women displaying much greater odds of clinical levels of GHQ-12 scores than men when intense caregiving.

### What is already known on this topic

Our results align with longitudinal evidence on the mental health effects of informal care.^5–7^^,^^23^ Consistent with Lacey et al. (2019), the distribution of informal care was gendered, with women undertaking care for more hours than men. Furthermore, the observed effects of informal care on mental health scores were only present for women.^5^ Aligned with Bom et al. (2019), who posited that the amount of care provided is an important source of variation in the health effects of informal care, women were at the highest risk of poor general mental health when providing intense caregiving. These findings highlight the need for carer support services to provide sustained support to meet the needs of caregivers, as stipulated in section 20 of the Caring Act 2014.^24^ While research is needed to understand the forms of support most effective and needed among carers, this support should be delivered holistically, accounting for the social context in which caregiving takes place and the complexity of the caregiving role.^16^ Carer support should be considered essential in managing future pandemics to ensure informal carers’ demands do not surpass their ability to cope.

While the overall population returned to some form of normal following lockdowns/confinement, informal carers likely remained isolated to protect the wellbeing of their families.^4^^,^^16^ Therefore, navigating and continuing to navigate the challenges related to the morbidities of the care recipient and their risk of developing severe symptoms of COVID-19 may compound the existing mental health effects of informal care. Recent findings on the impact of COVID-19 on carers suggest that many informal carers in the UK were excluded from support.^16^ Moreover, delays in diagnoses and specialized health care meant that informal carers and their families could not access the necessary information to address the needs of their caring recipient when their conditions were new, increasing the distress related to the caregiving role.^16^ In the context of the current adult social care reform, it is important to understand future challenges related to the COVID-19 pandemic. Future research should examine the extent to which caregivers’ physical and mental resilience may be impacted by post-viral sequalae, such as long COVID—experienced by the caregiver and recipient. Further research should also assess the impact of COVID-19 on the mental health of caregivers of people with conditions that may be accelerated by COVID-19, including dementia.

### What this study adds

While several papers investigating unpaid caregiving using Understanding Society emerged over the past 2 years,^11^^,^^12^^,^^25^ the present paper adds key information to the current evidence base. While other studies examined impacts of caregiving on mental health relatively early in the pandemic,^10^^,^^12^^,^^25^ our paper provides an overview of the mental health impact of informal care during the early and late stages of the pandemic. Moreover, we disaggregate informal carers into intense caregivers (caring for 20+ hours/week) and non-intense caregivers (1–19 hours/care week) providing evidence of a dose–response relationship. This knowledge is important for identifying informal carers at the greatest risk of poor mental health outcomes and informing targeted approaches for social care support. Compared with Costi et al. (2023), who focus on out-of-home carers, our paper extends the evidence base to those caring for someone within their households and elucidates the gendered effects of informal care.

Our findings indicate mental health changes for women associated with changes in their caregiving roles. Interestingly, women with intense caregiving demands displayed greatly increased odds of experiencing clinical levels of GHQ-12, compared to when not caregiving. This paper highlights the imperative for policymakers to recognize the contribution of informal carers and provide adequate support strategies to decrease the psychological burden of informal care, especially among women. Interventions and policies that aim to decrease the psychological strains of informal care must attend to the gender dynamics in caregiving. Pre-pandemic evidence indicates that women shoulder up to 80% of informal caregiving roles^26^ with clear impacts on their mental health.^27^ There is some concern that, as the population returns to normal, gender inequalities will be exacerbated due to gender normative roles and expectations.^28^^,^^29^ Accordingly, it is possible that post-COVID caregiving will disproportionately be borne by women in alignment with these gender normative arrangements. Therefore, the mental health impacts of informal care observed among women may persist over the coming years. Future research should continue to examine these gendered effects and identify means to ameliorate them.

### Limitations of this study

Considering that we investigated mental health changes due to caregiving transitions, our results only reflect the short-term effects of informal care. In addition, we could not account for time changes in the family structure due to living with an ill family member. Since people living with an ill family member show higher levels of distress than those with a different living arrangement and the caring demands of home carers are higher than in other carers,^18^ this may overestimate the observed effects. As such, accounting for family illness becomes instrumental when disentangling the mental health impact of caring for someone (‘caregiving effect’) from the one of caring about someone (‘family effect’).^17^ We partially controlled for this by accounting for time variations in living arrangements (e.g. living with an elderly relative). Also related to confounding, it is worth noting that the employment categories defined in this paper may not reflect the contextual labour dynamics during the pandemic and are likely inclusive of income and employment trajectories situated in the causal pathway between informal care and mental health. Future research should explore the role of different working arrangements (e.g. working from home, being an essential/key worker), on the mental health impact of informal care.

Another limitation is related to the use of self-reported measures. This limitation could affect our estimates in two ways. First, since participants were unaware of the aims of this study, there is a risk of non-differential measurement error of mental health, which could bias the estimates towards the null. Second, the estimated mental health effects of informal care may be subjected to common method bias, where exposed participants (e.g. informal carers) overstate their exposure and mental health symptoms, likely overestimating the observed results. We also identified a potential risk of selection bias, informal carers with clinical levels of depression and anxiety could be lost to follow-up at higher rates than non-carers and healthy participants, biassing our estimates towards the null. We minimized this risk by re-running our analyses in an imputed sample and using population weights.

Lastly, we could not restrict the analysis to new caregivers. This is important because the mental health strains of caregiving do not necessarily end when the caregiving role ceases. For example, caregiving may end when the person being cared for dies. Research on caregivers’ bereavement shows that the psychological impact of losing a family member is largest among caregivers than in any other population group.^30^ Therefore, our reference category (0 hours of weekly care) could include people who moved out of caregiving due to the loss of their caring recipient (or a decline in their health), potentially attenuating the observed effects.

Despite the limitations, most of which will likely underestimate the effects of informal caregiving, this paper has many strengths. First, we used a representative sample of the UK population, increasing the generalizability of our findings. Second, we used a highly validated mental health measure, reducing the risk of measurement error of the outcome. Third, we applied a robust approach to assess causation, minimizing the risk of measured and unmeasured time-invariant confounding factors such as cultural background, personality and temperament by allowing participants to act as their control. This approach also reduces the risk of common method bias since individual characteristics—controlled for in within-person analysis—are important drivers of misreporting.

## Supplementary Material

Supplementary_files_fdad193

## Data Availability

Data from Understanding Society can be accessed through the UK Data Service (https://ukdataservice.ac.uk/). Data specific to this paper can be requested from the corresponding author with prior approval from the data custodians.

## Notes

**Ludmila Fleitas Alfonzo**, PhD student

**Dr Yamna Taouk**, Research Fellow

**Dr Eric Emerson**, Emeritus Professor

**Dr Tania King**, Principal Research Fellow

## References

[1] Department of Health and Social Care . How Can We Improve Support for Carers? Government Response to the 2016 Call for Evidence [Internet]. London: DHSC, 2018 [cited 2022 Aug 2]. Available from: https://assets.publishing.service.gov.uk/government/uploads/system/uploads/attachment_data/file/713695/response-to-carers-call-for-evidence.pdf.

[2] Deparment of Work and Pensions . Family Resources Survey: Financial Year 2018 to 2019 [Internet]. London: Department of Work and Pensions, 2020 [updated 2020 Mar 26; cited 2022 Mar 7]. Available from: https://assets.publishing.service.gov.uk/government/uploads/system/uploads/attachment_data/file/874507/family-resources-survey-2018-19.pdf.

[3] Evandrou M, Falkingham J, Qin M et al. Older and ‘Staying at Home’ During Lockdown: Informal Care Receipt During the COVID-19 Pandemic amongst People Aged 70 and Over in the UK. Southampton: ESRC Centre for Population Change. 2020.

[4] Carers UK . Caring Behind Closed Doors. Forgotten Families in the Coronavirus Outbreak [Internet]. London: Carers UK, 2020 [updated c2020; cited 2022 Jul 26]. Available from: https://www.carersuk.org/images/News_and_campaigns/Behind_Closed_Doors_2020/Caring_behind_closed_doors_April20_pages_web_final.pdf.

[5] Lacey RE, McMunn A, Webb E. Informal caregiving patterns and trajectories of psychological distress in the UK Household Longitudinal Study. Psychol Med 2019;49(10):1652–60. 30205848 10.1017/S0033291718002222 PMC6601356

[6] Kaschowitz J, Brandt M. Health effects of informal caregiving across Europe: a longitudinal approach. Soc Sci Med 2017;173:72–80. 27930918 10.1016/j.socscimed.2016.11.036

[7] Bom J, Stöckel J. Is the grass greener on the other side? The health impact of providing informal care in the UK and the Netherlands. Soc Sci Med 2021;269:113562. 33339683 10.1016/j.socscimed.2020.113562

[8] Bom J, Bakx P, Schut F et al. The impact of informal caregiving for older adults on the health of various types of caregivers: a systematic review. Gerontologist 2018;59(5):e629–e42. 10.1093/geront/gny137 PMC6850889 30395200

[9] Pierce M, McManus S, Hope H et al. Mental health responses to the COVID-19 pandemic: a latent class trajectory analysis using longitudinal UK data. Lancet Psychiatry 2021;8(7):610–9. 33965057 10.1016/S2215-0366(21)00151-6 PMC9764381

[10] Whitley E, Reeve K, Benzeval M. Tracking the mental health of home-carers during the first COVID-19 national lockdown: evidence from a nationally representative UK survey. Psychol Med 2021;53(3):1–31. 34108060 10.1017/S0033291721002555 PMC8245331

[11] Costi C, Hollingsworth B, O'Sullivan V et al. Does caring for others affect our mental health? Evidence from the COVID-19 pandemic. Soc Sci Med 2023;321:115721. 36827903 10.1016/j.socscimed.2023.115721 PMC9872568

[12] Gallagher S, Wetherell MA. Risk of depression in family caregivers: unintended consequence of COVID-19. BJPsych Open 2020;6(6):e119. 33040759 10.1192/bjo.2020.99 PMC7550870

[13] Helson H . Adaptation-Level Theory: An Experimental and Systematic Approach to Behavior. Harper and Row: New York, 1964.

[14] Pearlin LI . The life course and the stress process: some conceptual comparisons. J Gerontol B Psychol Sci Soc Sci 2010;65b(2):207–15. 20022925 10.1093/geronb/gbp106 PMC2821941

[15] Howatson-Jones L, Coren E. Scoping the assessment needs of young carers of adults with a long term condition. J Nurs Care 2013;2:1–4.

[16] Onwumere J, Creswell C, Livingston G et al. COVID-19 and UK family carers: policy implications. Lancet Psychiatry 2021;8(10):929–36. 34537103 10.1016/S2215-0366(21)00206-6 PMC8445736

[17] Bobinac A, van Exel NJA, Rutten FFH et al. Caring for and caring about: disentangling the caregiver effect and the family effect. J Health Econ 2010;29(4):549–56. 20579755 10.1016/j.jhealeco.2010.05.003

[18] Bom J, Bakx P, Schut F et al. Health effects of caring for and about parents and spouses. J Econ Ageing 2019;14:100196.

[19] Institute for Social Economic Research . Understanding Society COVID-19 User Guide. Version 10.0, October 2021. Colchester: University of Essex, 2021.

[20] Mindell J, Biddulph JP, Hirani V et al. Cohort profile: the health survey for England. Int J Epidemiol 2012;41(6):1585–93. 22253315 10.1093/ije/dyr199

[21] Goldberg DP, Gater R, Sartorius N et al. The validity of two versions of the GHQ in the WHO study of mental illness in general health care. Psychol Med 1997;27(1):191–7. 9122299 10.1017/s0033291796004242

[22] Gunasekara FI, Richardson K, Carter K et al. Fixed effects analysis of repeated measures data. Int J Epidemiol 2013;43(1):264–9. 24366487 10.1093/ije/dyt221

[23] Rafnsson SB, Shankar A, Steptoe A. Informal caregiving transitions, subjective well-being and depressed mood: findings from the English Longitudinal Study of ageing. Aging Ment Health 2017;21(1):104–12. 26404725 10.1080/13607863.2015.1088510 PMC5582155

[24] Care Act 2014 [Internet] . 2014 [updated 2021 Jul 7; cited 2023 Jun 6]. Available from: https://www.legislation.gov.uk/ukpga/2014/23/section/20/enacted.

[25] Xue B, McMunn A. Gender differences in unpaid care work and psychological distress in the UK Covid-19 lockdown. PloS One 2021;16(3):e0247959. 33662014 10.1371/journal.pone.0247959 PMC7932161

[26] Cascella, Carbó GF, García-Orellán R. Burden and gender inequalities around informal care. Investigación y educación en enfermería 2020;38(1):e(10). 10.17533/udea.iee.v38n1e10. PMC7871478 32124578

[27] Ervin J, Taouk Y, Fleitas Alfonzo L et al. Longitudinal association between informal unpaid caregiving and mental health amongst working age adults in high-income OECD countries: a systematic review. eClinicalMedicine 2022;53:101711. 36353526 10.1016/j.eclinm.2022.101711 PMC9637877

[28] King T, Hewitt B, Crammond B et al. Reordering gender systems: can COVID-19 lead to improved gender equality and health? The Lancet 2020;396(10244):80–1. 10.1016/S0140-6736(20)31418-5 PMC7304958 32569582

[29] King TL, Maheen H, Taouk Y et al. Precarious work and the Covid-19 pandemic: the need for a gender equality focus. BMJ. (Clinical research ed.), 2023;380:e072872. 10.1136/bmj-2022-072872. 36717131

[30] Moriarty J, Maguire A, O’Reilly D et al. Bereavement after informal caregiving: assessing mental health burden using linked population data. Am J Public Health 2015;105(8):1630–7. 26066918 10.2105/AJPH.2015.302597 PMC4504303

