# Supplementary material for: Impact of informal care on the mental health of caregivers during the COVID-19 pandemic
Source: J Public Health (Oxf). 2023 Oct 2;45(4):e668–76. doi: 10.1093/pubmed/fdad193 (PMC10687605; doi:10.1093/pubmed/fdad193)
Supplement: Supplementary_files_fdad193 [file supplementary_files_fdad193.docx]

Supplementary files

Contents

[Supplementary methods 2](#_Toc142411062)

[Participants 2](#_Toc142411063)

[Informal care – survey question in COVID-19 surveys 2](#_Toc142411064)

[GHQ-12 description and categorisation. 2](#_Toc142411065)

[Ascertainment of gender 3](#_Toc142411066)

[Statistical analysis 3](#_Toc142411067)

[Multiple imputation – details. 3](#_Toc142411068)

[Supplementary results 4](#_Toc142411069)

[Fixed effects regression analyses 4](#_Toc142411070)

[Supplementary table 1. Informal care transition probabilities. 5](#_Toc142411071)

[Supplementary Table 2. Between and within distribution of informal care across participants with complete data (n= 16,180, obs=61,195) 5](#_Toc142411072)

[Supplementary table 3. Mental health effects of within person changes in informal care for women in the imputed sample. 6](#_Toc142411073)

[Supplementary table 4. Mental health effects of within person changes in informal care for men in the imputed sample. 7](#_Toc142411074)

[Supplementary table 5. Mental health effects of within person changes in informal care for all participants in the imputed sample. 8](#_Toc142411075)

[References 9](#_Toc142411076)

# Supplementary methods

## Participants

The main survey sample includes a general sample from Great Britain and Northern Ireland, an Ethnic Boost sample and an Immigrant Boost sample to ensure the representativeness of people from culturally diverse backgrounds. Understanding Society COVID-19 surveys are an initiative of the Institute for Social and Economic Research at the University of Essex and the Health Foundation. Data is available from the UK Data Service.

In wave 1, about 40,000 households were recruited to participate in Understanding Society, yielding an initial sample of 50,199 individual respondents. In April 2020, a COVID-19 survey was implemented to a sub sample of 17,761 participants to document the impact of the COVID-19 pandemic in the UK. These respondents were selected from participants to waves 8 or 9 of the main survey (n=42,221). A total of 13,764 participants responded to the follow-up July 2020 COVID-19 survey.(21) All data was collected through online surveys and computer-assisted interviews.

## Informal care – survey question in COVID-19 surveys

In order to identify informal carers, participants were asked the following question *“Is there anyone living with you who is sick, disabled or elderly whom you look after or give special help to (for example, a sick, disabled or elderly relative, husband, wife or friend etc)”*. Those answering yes were then asked to indicate the number of weekly hours of care they provided. The use of hours of care over a binary classification of caring status was preferred because it provides information about the intensity of caregiving activities.(1) This an important distinction because informal care disproportionately affects people undertaking extensive caregiving demands. (1)

## GHQ-12 description and categorisation.

The GHQ-12 evaluates general levels of mental distress through twelve items indicating experiences of mental distress in the previous fortnight.(2) Participants’ responses included the following options: 0 “Not at all”, 1 “No more than usual”, 2 “Rather more than usual” and 3 “More than usual”. Responses to all items were combined to create total scores of GHQ-12 (ranging from 0-36). These scores were analysed as a continuous measure, with greater scores indicating poorer mental health. A binary classification of GHQ-12 was also examined to assess the effect of informal care on clinically significant levels of psychological distress. Total scores of GHQ-12 ranging from 0-12 were generated by recoding responses to “not at all” and “no more than usual” as 0 and responses to “rather more than usual” and “much more than usual” as 1. We used a cut-off of 4 or more to indicate probable cases of clinical levels of mental distress as, this is formally described as an indicator of GHQ caseness.(3, 4) Participants were categorised into two groups, those falling under the cut-off (0 or no probable clinical levels of mental distress) and those with scores of 4 and above (1 or probable clinical levels of mental distress).

## Ascertainment of gender

Since gender identification questions were not asked in the COVID-19 surveys, gender was derived from participant’s self-reports of sex and categorised as a binary variable (men or women). It is also important to clarify that although we acknowledge that gender is non-binary, the number of participants who did not identify as males or females was small (n<10) and were therefore excluded from our analyses.

## Statistical analysis

As changes are examined in an individual level, each participant acts as their own control, allowing control for time-invariant confounding factors such as ethnicity and other individual characteristics.(5) All analyses were adjusted to account for changes in time (wave), and the covariates listed the methods section.

## Multiple imputation – details.

All covariates and the mental health variable were included in the imputation model with the following ancillary variables, which were extracted from wave 4 of the COVID-19 survey: key/essential COVID-19 worker, change in residential address and COVID-19 infection. These were identified as probable reasons for missing and included in the imputation model to meet the assumption of missing at random (MAR) as specified by Rubin and Schenker (6)

# Supplementary results

## Fixed effects regression analyses

Within-person changes in hours of informal care were associated with an increase of 0.36 (95%CI: -0.18, 0.90) in GHQ-12 when caring for 1-19 hours/week and an increase of 0.77 (95%CI: 0.12, 1.43) caring for ≥20 hours. In terms of GHQ caseness, as compared to not caregiving, participants showed an increase of 1.21 in odds of poor levels of distress when caring for 1-19 hours/week and a change of 1.75 when caring for ≥20 hours. These estimates, however, demonstrated high uncertainty levels, with 95% CIs ranging from 0.75 to 1.94 when caring for 19 hours/week, and 0.98 to 3.14 when caring for ≥20 hours.

# Supplementary table 1. Informal care transition probabilities.

|  | Final values | | |
| --- | --- | --- | --- |
|  | **0 hours of care** | **1-19 hours of care** | **≥20 hours of care** |
| *Initial values (36,873)* | n (%) | n (%) | n (%) |
| **0 hours of care** *(35,036)* | *34,568 (98.7)* | 310 (0.88) | 158 (0.45) |
| **1-19 hours of care (869)** | 264 (30.4) | *483 (55.6)* | 122 (14.0) |
| **≥20 hours of care (968)** | 147 (15.2) | 82 (8.47) | *739 (76.3)* |

Note: Stata output from “xttrans”

# Supplementary Table 2. Between and within distribution of informal care across participants with complete data (n= 16,180, obs=61,195)

|  | **Overall** | | **Between** | | **Within** |
| --- | --- | --- | --- | --- | --- |
|  | n | % | n | % | % |
| **0 hours of care** | 47,839 | 94.9 | 13,082 | 96.5 | 98.0 |
| **1-19 hours of care** | 1,202 | 2.38 | 641 | 4.73 | 52.5 |
| **≥20 hours of care** | 1,389 | 2.75 | 585 | 4.32 | 69.3 |
| **Total** | 50,430 | 100 | 14,308 | 105.4 | 94.8 |

Note: Stata output from “xttab”

# Supplementary table 3. Mental health effects of within person changes in informal care for women in the imputed sample.

|  | **Model 1** | **Model 2** |
| --- | --- | --- |
|  | *n=8,131 obs=29,412* | *n=7,660 , obs=22,496* |
| **GHQ-12 (scores)** | **β (95%CI)** | **β (95%CI)** |
| 0 hours of care | *ref* | *ref* |
| 1-19 hours of care | 0.50 (-0.11, 1.10) | 0.72 (-0.03, 1.46) |
| ≥20 hours of care | 1.03 (0.11, 1.95) | 0.90 (-0.25, 2.05) |
| **GHQ-12 (caseness)** | **OR (95%CI)** | **OR (95%CI)** |
| 0 hours of care | *ref* | *ref* |
| 1-19 hours of care | 1.31 (0.73, 2.35) | 1.92 (0.92, 3.99) |
| ≥20 hours of care | 2.32 (1.15, 4.67) | 2.19 (0.92, 5.21) |

Model 1: adjusted for age, household structure (living with a partner, a children aged <5 years and living with an elderly) and wave of COVID-19 survey.

Model 2: adjusted for variables in model 1, employment and quintiles of weekly household income.

# Supplementary table 4. Mental health effects of within person changes in informal care for men in the imputed sample.

|  | **Model 1** | **Model 2** |
| --- | --- | --- |
|  | *n=6,090 , obs=22,479* | *n=5,836, obs=18,239* |
| **GHQ-12 (scores)** | **β (95%CI)** | **β (95%CI)** |
| 0 hours of care | *ref* | *ref* |
| 1-19 hours of care | 0.20 (-0.66, 1.06) | 0.41 (-0.63, 1.45) |
| ≥20 hours of care | 0.23 (-0.50, 0.96) | -0.11 (-1.00, 0.78) |
| **GHQ-12 (caseness)** | **OR (95%CI)** | **OR (95%CI)** |
| 0 hours of care | *ref* | *ref* |
| 1-19 hours of care | 0.91 (0.42, 2.01) | 0.96 (0.32, 2.90) |
| ≥20 hours of care | 0.73 (0.28, 1.91) | 0.70 (0.21, 2.28) |

Model 1: adjusted for age, household structure (living with a partner, a children aged <5 years and living with an elderly) and wave of COVID-19 survey.

Model 2: adjusted for variables in model 1, employment and quintiles of weekly household income.

# Supplementary table 5. Mental health effects of within person changes in informal care for all participants in the imputed sample.

|  | **Model 1** | **Model 2** |
| --- | --- | --- |
|  | *n=14,223 obs=51,922* | *n=13,498; obs=40,754* |
| **GHQ-12 (scores)** | **β (95%CI)** | **β (95%CI)** |
| 0 hours of care | *ref* | *ref* |
| 1-19 hours of care | 0.36 (-0.18, 0.91) | 0.59 (-0.07, 1.25) |
| ≥20 hours of care | 0.80 (0.14, 1.46) | 0.59 (-0.27, 1.45) |
| **GHQ-12 (caseness)** | **OR (95%CI)** | **OR (95%CI)** |
| 0 hours of care | *ref* | *ref* |
| 1-19 hours of care | 1.21 (0.75, 1.96) | 1.51 (0.82, 2.78) |
| ≥20 hours of care | 1.79 (0.99, 3.23) | 1.62 (0.79, 3.31) |

Model 1: adjusted for age, household structure (living with a partner, a children aged <5 years and living with an elderly) and wave of COVID-19 survey.

Model 2: adjusted for variables in model 1, employment and quintiles of weekly household income.

# References

1. Bom J, Bakx P, Schut F, van Doorslaer E. The Impact of Informal Caregiving for Older Adults on the Health of Various Types of Caregivers: A Systematic Review. The Gerontologist. 2018; 59:e629-e42.

2. Pevalin DJ. Multiple applications of the GHQ-12 in a general population sample: an investigation of long-term retest effects. Social psychiatry and psychiatric epidemiology. 2000; 35:508-12.

3. Goldberg DP, Gater R, Sartorius N, Ustun TB, Piccinelli M, Gureje O, et al. The validity of two versions of the GHQ in the WHO study of mental illness in general health care. Psychological medicine. 1997; 27:191-7.

4. Morris S, Earl K, Neave A. Health survey for England 2016: well-being and mental health. London: Health. 2017.

5. Gunasekara FI, Richardson K, Carter K, Blakely T. Fixed effects analysis of repeated measures data. International Journal of Epidemiology. 2013; 43:264-9.

6. Rubin DB, Schenker N. Multiple imputation in health‐are databases: An overview and some applications. Statistics in medicine. 1991; 10:585-98.
